# 

**Published:** 1895-06

**Authors:** 


### TABLE OF CONTENTS.

ORIGINAL COMMUNICATIONS. PAGE

A Method of inserting Gold Fillings with the Use of Hand-Burnishers, as practised exclusively for Seventeen Years. By DR. HENRY F. LIBBY, Boston, Mass. . . . . . . . . . . . . . . . . . . . . . . . . . 325

The Scientific Spirit and the Ethics of Dental Practice. By DR. C. J ESSIG, Philadelphia . . . . . . . . . . . . . . . . . . . . . . . . . 343

Plastics as a Bar to Bacteria. By JOSEPH HEAD, M.D., D.D.S., Philadelphia . . . . . . . . . . . . . . . . . . . . . . . . . . . 349

Glass Fillings, Jacket Crowns, Amalgam, Gold Caps, Bridges, etc.—A Scrap of their History. By WILLIAM H. TRUEMAN, Philadelphia . . . 353

REPORTS OF SOCIETY MEETINGS.

American Academy of Dental Science . . . . . . . . . . . . . . . . . . 358

Odontological Society of Pennsylvania . . . . . . . . . . . . . . . . . 369

Academy of Stomatology . . . . . . . . . . . . . . . . . . . . . . . . 372

EDITORIAL.

A Greater Need for Professional Dignity . . . . . . . . . . . . . . . . 379

Asbury Park and the Conventions . . . . . . . . . . . . . . . . . . . 381

OBITUARY.

Dr. John J. R. Patrick . . . . . . . . . . . . . . . . . . . . . . . . 382

Dr. Ludwig Adolph Weil . . . . . . . . . . . . . . . . . . . . . . . . 383

CURRENT NEWS.

Chicago Dental Society . . . . . . . . . . . . . . . . . . . . . . . . 384

Missouri State Dental Association . . . . . . . . . . . . . . . . . . 384

National Association of Dental Examiners . . . . . . . . . . . . . . . 385

Pennsylvania State Dental Examining Board . . . . . . . . . . . . . . 385

New Jersey State Dental Society . . . . . . . . . . . . . . . . . . . . 385

American Dental Association . . . . . . . . . . . . . . . . . . . . . 386

Massachusetts Dental Society . . . . . . . . . . . . . . . . . . . . . 386

Woman's Dental Association . . . . . . . . . . . . . . . . . . . . . . 387

SELECTIONS.

Diiodoform . . . . . . . . . . . . . . . . . . . . . . . . . . . . . . 387

